# On the Treatment of Scabies

**Published:** 1849-03

**Authors:** 


*On the Treatment of Scabies.* By G. Corfe, Esq.—Mr. Corfe states that he rigorously pursues the following plan with a patient affected with itch:—

We provide him with old soiled linen and a worn out sheet; and each morning and evening he is ordered to make a good lather of yellow soap in his hands, and thus dip them wet into a basin of sifted or fine sand, and assiduously rub every part of the body on which the slightest trace of a vesicle exists. Having performed this ablution until the skin tingles smartly, he wipes himself dry, and then rubs the common ung. sulphuris firmly into the itchy parts. He is then enveloped in the winding sheet, and has a pair of old gloves on his hands, and he is left till night, when the same operation is pursued, and repeated daily until the fourth day, when he is ordered to indulge (and a great indulgence it is) in a warm bath, where he again lathers his body in plain soap and water, puts on fresh linen, and is provided with clean sheets, and the cure is from thence invariably effected. The vesicle of course is broken by the friction of the sand and soap; the acarus is exposed, and this ectozoon receives his death-blow by the inunction of the sulphur, which is oftentimes not accomplished by the mere application of sulphur ointment alone. The use of sand-soap balls is more elegant, though not more efficacious.—*Medical Times.*

